# Vascular Choroidal Alterations in Uncomplicated Third-Trimester Pregnancy

**DOI:** 10.3390/tomography8050218

**Published:** 2022-10-18

**Authors:** Jan A. M. Sochurek, Michael Gembicki, Salvatore Grisanti, Mahdy Ranjbar

**Affiliations:** 1 Laboratory for Angiogenesis and Ocular Cell Transplantation, Ratzeburger Allee 160, 23538 Lübeck, Germany; 2 Department of Gynecology and Obstetrics, University of Lübeck, Ratzeburger Allee 160, 23538 Lübeck, Germany; 3 Department of Ophthalmology, University of Lübeck, Ratzeburger Allee 160, 23538 Lübeck, Germany

**Keywords:** EDI-OCT, OCTA, choroid, pregnancy, biomarker

## Abstract

(1) Purpose: To evaluate the anatomy and perfusion of choroidal substructures in third-trimester pregnant women using optical coherence tomography (OCT) and OCT angiography (OCTA) imaging. (2) Methods: In this cross-sectional study, women in their third trimester of uncomplicated pregnancy and non-pregnant age-matched women were recruited. Participants underwent enhanced depth imaging (EDI) OCT and OCTA. Subfoveal choroidal thickness (SFCT), as well as choroidal sublayer perfusion, were compared between groups. (3) Results: In total, 26 eyes of 26 pregnant and 26 eyes of 26 non-pregnant women were included. The median age in both groups was 29 years. The median SFCT was 332 (211–469) µm in the pregnant group and 371.5 (224–466) µm in the non-pregnant cohort (*p* = 0.018). The median choriocapillaris perfusion (CCP) was significantly lower in the pregnant group (46% vs. 48%, *p* = 0.039). Moreover, Haller’s layer perfusion correlated significantly with mean arterial pressure in non-pregnant women (CC = 0.430, *p* = 0.028) but not in pregnant ones (CC = 0.054, *p* = 0.792). (4) Conclusions: SFCT was found to be thinner and CCP was lower in third-trimester pregnant women. Hormonal changes during pregnancy and consecutive impacts on autoregulation of small choroidal vessels might play an important role. Therefore, altered choroidal measurements during third-trimester pregnancy should be carefully evaluated as, to some extent, it could be a normal physiological change.

## 1. Introduction

Pregnancy is accompanied by vast physiological changes throughout the body. Continuous adjustments are made in the hematological, vascular, metabolic, and immunological systems during this time [1]. Hemodynamic changes in pregnancy include an increase in plasma volume, cardiac output, peripheral vasodilation, and reduction in systemic vascular resistance [2]. The decrease in peripheral resistance is mainly driven by a considerable upregulation of nitric oxide through the rise of estrogen (10-fold) and progesterone (5-fold), which reach their maximum during the third trimester [3]. This reaction prevents an increase in mean arterial pressure (MAP) and supports the redistribution of blood flow to organs of special interest, such as the uterus and the placenta [1,2,3]. Therefore, it can be expected that these systemic changes might also have an impact on ocular perfusion.

The choroid plays a significant role in the physiology of the eye as it supplies blood to vital ocular structures. It is often key in the pathogenesis of various ocular diseases [4]. Furthermore, it can be severely affected by pathological conditions during pregnancy, such as pre-eclampsia and central serous chorioretinopathy (CSC) [5]. Imaging of the choroidal vasculature during pregnancy has been limited due to concerns regarding the use of mydriatic eye drops and fluorescein dye. However, optical coherence tomography (OCT) and technical upgrades such as enhanced depth imaging (EDI), as well as OCT angiography (OCTA), cleared that obstacle as they are non-invasive and do not require pupillary dilation [6,7,8]. OCT- and OCTA-based imaging of the choroid revolutionized our pathophysiological understanding of various diseases, and choroidal metrics such as subfoveal choroidal thickness (SFCT) have been suggested as potential biomarkers in this regard [9,10].

To explore the significance of the choroid and to utilize its characteristics as a reliable biomarker, normative values are needed. It has been suggested that choroidal metrics vary with age, axial length (AL), MAP, and the time of day [11,12]. In light of the dramatic systemic changes during pregnancy, an impact on choroidal circulation and structure may be expected. Several studies have explored SFCT in pregnant women, but results have been inconclusive [13]. However, data regarding vascular changes have been sparse to date, as only one group reported no difference in choriocapillaris perfusion (CCP) of uncomplicated third-trimester pregnant women compared with non-pregnant controls [14].

We previously reported on choroidal sublayer perfusion in various cohorts, evaluating Sattler’s layer perfusion (SLP) and Haller’s layer perfusion (HLP) in addition to CCP [11,15,16]. Therefore, in this study, we sought to investigate each choroidal sublayer perfusion in uncomplicated third-trimester pregnancy. This information can be used to determine whether abnormal choroidal vascular measurements are potential indicators for complications during pregnancy.

## 2. Methods

This cross-sectional study was conducted following the Declaration of Helsinki as well as relevant local and national guidelines. Approval by the ethics committee of the University of Lübeck, Germany (vote reference #18-239) was given, and written informed consent was obtained from each participant before enrolment in the study.

Inclusion criteria for the pregnancy group included healthy women with uncomplicated pregnancies in the third trimester (>28 weeks gestational age). Subjects were excluded if they had pre-existing medical conditions, such as diabetes mellitus or hypertension and/or pregnancy complications, such as gestational diabetes or preeclampsia. A control group of age-matched non-pregnant women without significant past medical or ocular history was included for comparative analysis. Additional exclusion criteria for both groups included the presence of any ocular disease and/or media opacity. The refractive error needed to be within ±3 spherical diopters as well as less than −2 cylindrical diopters, while individuals with more than ±3 diopters spherical equivalent were also excluded. All participants underwent extended examinations, including the assessment of blood pressure (BP), refraction, best-corrected visual acuity (BCVA) in Snellen, intraocular pressure (IOP), AL, and slit-lamp biomicroscopy including indirect fundoscopy.

Firstly, it was verified that none of the participants had consumed caffeine or had taken any kind of medication such as analgesics for at least 24 h. After a rest period of 30 min, in which participants were placed in a quiet room with ambient light and were asked to avoid using their tablet or mobile phone, all images were taken by a single trained operator under standardized conditions, such as normal lighting, and without prior pupil dilatation using the CIRRUS 5000 OCT device with AngioPlex software (Version 11.0; Carl Zeiss Meditec, Inc., Dublin, CA, USA). The device has an A-scan rate of 68,000 scans per second and an 840 nm light source with a bandwidth of 90 nm. OCTA images were generated using the OMAG algorithm, and an auxiliary real-time line scan ophthalmoscope (LSO) reduced motion artifacts. Similar to our previous reports, volumetric EDI-OCT (10 × 10 mm^2^) and OCTA (3 × 3 mm^2^) scans of the posterior pole of both eyes were captured [8,11,15]. As recommended by the guidelines on OCTA imaging, only scans with a signal strength ≥ 8, centered on the fovea, and without motion, segmentation, and projection artifacts were included [17]. To avoid inaccuracies due to diurnal changes in perfusion, OCTA images were acquired between 8 a.m. and noon.

Subfoveal choroidal thickness was manually measured in EDI-OCT scans at a single point below the fovea, extending from the bottom of the hyper-reflective layer corresponding to Bruch’s membrane to the hyper-reflective layer at the sclera–choroidal junction, as previously reported [11].

**Figure 1:**
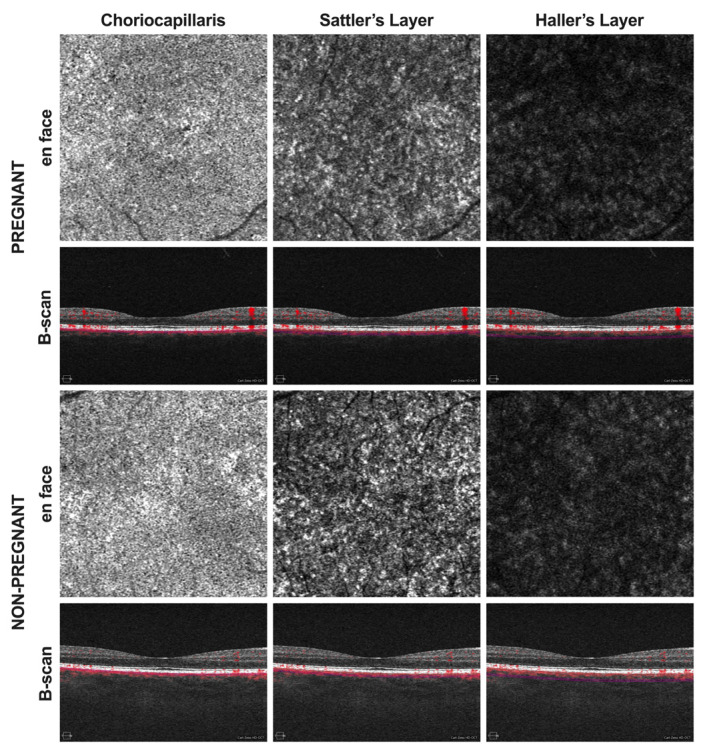
Optical coherence tomography angiography (OCTA) images were acquired in pregnant as well as non-pregnant women. En face angiograms and corresponding B-scans of the choriocapillaris, Sattler’s layer, and Haller’s layer were generated using the CIRRUS 5000 OCT device with AngioPlex software. En face images were then processed for quantitative analysis using ImageJ.

**Figure 2:**
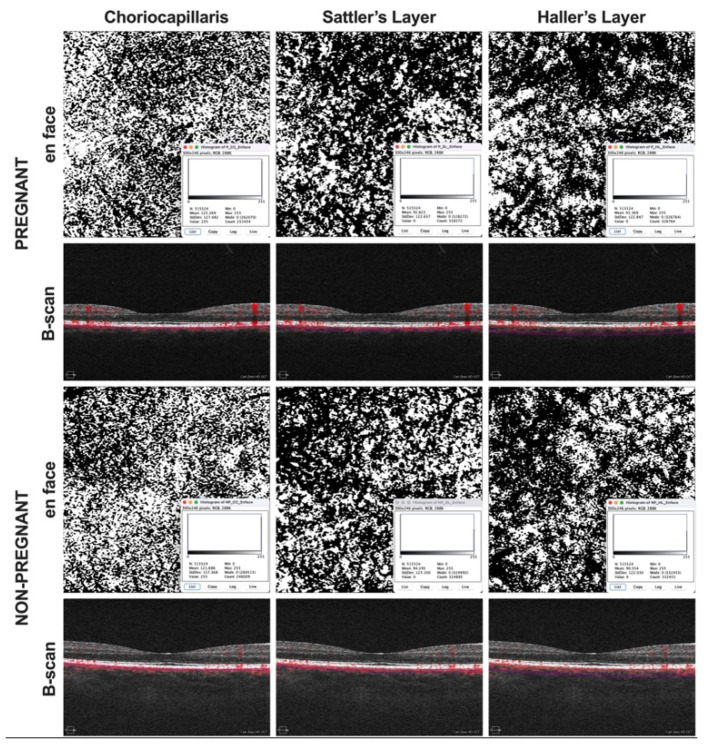
Binarization of angiograms. Images were exported into ImageJ and binarized using the Otsu method. The percentage of white (CC) or black pixels (SL and HL) was considered an indirect measure of the choroidal vascular flow area.

Optical coherence tomography angiography data were manually segmented according to previous protocols to obtain 20 µm slabs of each choroidal sublayer (Figure 1) [11,15,16]. Each angiogram was then exported as an image into ImageJ (NIH, version 1.48b, Bethesda, MD, USA) and binarized using the command path Image > Adjust > Threshold > Auto to calculate the percentage of black and white pixels. Binarization was performed by the Otsu method, which is an automatic threshold selection from gray-level histograms. It has been suggested that the percentage of white (CC) or black pixels (SL and HL) is an indirect measure of the choroidal vascular flow area. Therefore, either the white or the black pixel value of the respective layer was calculated and divided by the whole scan area to determine CCP, SLP, and HLP (Figure 2).

The data were analyzed using IBM SPSS (Version 24.0, Chicago, IL, USA), and the figure was generated using Prism GraphPad (Version 9.0, La Jolla, CA, USA). Snellen BCVA was converted to the logarithm of the minimum angle of resolution (logMAR). The mean arterial pressure was calculated based on systolic and diastolic BP (MAP = 2/3 DBP + 1⁄3 SBP). The data were tested for normality using the Shapiro–Wilk test. Since the data were not normally distributed, the following non-parametric statistical tests were applied. To compare means between groups, the Mann–Whitney U test was used. In addition, the association between variables was measured by Spearman’s rank correlation. Values are reported as median (range), and a *p*-value of <0.05 was considered statistically significant.

## 3. Results

A total of 30 pregnant and 30 age-matched non-pregnant European women were recruited. However, due to OCTA image motion artifacts, four participants had to be excluded from each group. All pregnant subjects were uncomplicated, singleton pregnancies. The median gestational age of the pregnant women was 35 (32–40) weeks. Only one eye of each subject was selected for further analysis. Laterality was assigned by chance, which resulted in 14 of the study eyes being right and 12 being left in the pregnant group and 11 being right and 15 being left in non-pregnant controls. Demographics and clinical data of the enrolled subjects are shown in Table 1. There was no statistical difference in age between groups. Most importantly, significant parameters for OCT and OCTA evaluation such as AL, BCVA, IOP, and MAP were not significantly different (*p* > 0.05). Moreover, the median OCTA image quality was 10 (out of 10) in both groups.

Choroidal metrics are reported in Table 2. The median SFCT in pregnant subjects was 332 (211–469) µm, which was significantly thinner than in non-pregnant women, who had a median SFCT of 371.5 (224–466) µm (*p* = 0.018). Perfusion analysis of choroidal sublayers revealed a statistically significant difference, as CCP in the pregnant group was 46% (41–50%) compared with 48% (41–52%) in the control group (*p* = 0.039). However, neither SLP nor HLP showed any significant distinction between groups (*p* > 0.05).

Correlation analysis results are shown in Table 3 and Table 4. In the non-pregnant control group, HLP was significantly correlated with MAP (cc = 0.430, *p* = 0.028). Both CCP and SLP did not show any statistically significant correlation to MAP (*p* > 0.05). In pregnant women, there was no significant correlation between MAP and perfusion of any choroidal sublayer (*p* > 0.05). However, the pregnancy group demonstrated a positive correlation among the perfusion in all choroidal sublayers.

## 4. Discussion

In the present study, we compared choroidal sublayer perfusion between pregnant women in the third trimester and age-matched non-pregnant controls. This study confirms and expands previous research investigating choroidal metrics in pregnant women. To date, ophthalmological research was mostly conducted on retinal and choriocapillaris perfusion or general choroidal anatomy during pregnancy. Hence, to the best of our knowledge, this is the first study to report on perfusion of each choroidal sublayer in pregnant women using OCTA. The results of our analysis indicate that SFCT is thinner and, of all three choroidal vascular layers, only the choriocapillaris perfusion is significantly lower in third-trimester pregnant women compared with non-pregnant controls.

Pregnancy is a unique period, accompanied by considerable hormonal, hemodynamic, and cardiovascular changes [1]. The choroid is a primarily vascular and cavernous structure [18]. In the choroid, the flow per perfused volume is the highest of any other human tissue, and the choroidal vessels and capillaries contain about 85% of the overall ocular blood flow [19]. Therefore, its anatomy is mainly driven by ocular blood flow, which previous studies have shown to be altered in pregnant women [20,21]. Kara et al. reported the choroid to be significantly thicker (about 35 µm) in pregnant women than in the non-pregnant controls [22]. Correspondingly, Azuma et al. evaluated the subfoveal choroidal vascularity index, which was significantly higher in the pregnancy group [20]. By contrast, in the current study, the SFCT was significantly thinner in pregnant women. Characteristics of the different study populations might give us an explanation. Especially, gestational age is considerably distinctive (27 and 11 vs. 35 weeks), which is fairly important when considering the findings of Dadaci et al. that choroidal thickness decreases during the course of pregnancy [23]. On a hormonal level, estrogen, progesterone, and renin–angiotensin levels increase considerably during pregnancy [3]. While estrogens exert a vasodilatory effect on tissue perfusion, progesterone and renin–angiotensin have the opposite impact as they increase the resistance of the ophthalmic artery and its branches [24,25]. A pregnancy-induced imbalance between these vasoregulatory hormones might lead to either hyper- or hypoperfusion of the choroid or no changes at all. In contrast to our findings, Su et al. did not find any statistically significant differences in choroidal metrics when comparing third-trimester pregnant women to non-pregnant ones [14]. However, various trends were evident in their study, as mean SFCT was about 40 µm thinner and mean total subfoveal choroidal area, as well as subfoveal luminal area, were almost 10% smaller in pregnant women, which could be an indication of reduced vascular perfusion. Considering the sample size, their study (12 pregnant women) might have been slightly underpowered to elucidate statistical disparities. Moreover, they considered choroidal values of both eyes in some of their participants and did not adjust for inter-eye correlations, which from a statistical point of view is important. Finally, they used another OCTA device (Heidelberg Engineering vs. Zeiss) and a different thresholding algorithm (Phansalkar vs. Otsu) compared with us, which is essential as it usually does not allow comparability of results [26].

We previously reported on diurnal changes in choroidal sublayer perfusion and highlighted the importance of the time of day for comparability of OCTA measurements between groups but also in the same individual for longitudinal studies [11,16]. Therefore, we analyzed our participants during the same 4 h examination window (8:00–12:00), which other groups did not, as reported in their study limitations [14,27]. Furthermore, we previously demonstrated that while HLP usually correlates with MAP, CCP does not, and we suggested that the latter one is autoregulated by myogenic, neuronal, and hormonal contributors [11]. Thus, pregnancy-induced differences in choriocapillaris perfusion are not surprising. However, it is quite interesting that the positive correlation between MAP and HLP was not evident in pregnant women anymore, which could be due to local or systemic vasomodulatory processes.

Our study has some limitations. First, we did not look at the hormonal status of participants. As hormones have a significant impact on systemic vessel dynamics in terms of constriction and dilatation, evaluating their composition is important and should be considered [28]. In this regard, the menstruation cycle of non-pregnant women, which we did not take into consideration, is also important. Second, we did not fully take differences in time–activity patterns into account, as pregnant women tend to spend more time at home during the latter stages of pregnancy and, by doing so, might be more exposed to specific home-based factors and activities (e.g., flickering light from televisions) [29,30]. Third, the scanned area of 3 × 3 mm^2^ was not enough to suggest global differences of the choroid. A wider range of the examination area may be more conclusive. Fourth, by using a single OCTA device, our methodical approach is restricted, as perfusion values differ from device to device, depending on hardware and segmentation, as well as software algorithm, and it remains to be tested whether our findings can be validated using various commercially available OCTA devices. Fifth and finally, the sample size is relatively small, however, it is much bigger than comparable studies evaluating retinal and choriocapillaris perfusion during pregnancy using OCTA, which range between 10 to 16 participants [14,20,27]. We computed the required sample size based on preliminary data, which indicated the need for at least 18 values for each group to achieve a statistical power of more than 95%. By choosing only one eye randomly, we aimed at an evenly weighted impact of each participant.

In summary, using EDI-OCT and OCTA, we observed that SFCT and CCP were significantly different in uncomplicated third-trimester pregnant women compared with the eyes of age-matched controls. The knowledge of what direction normal pregnancy changes choroidal metrics is important and allows clinicians to assess choroidal measurements more precisely and use them as a biomarker for diseases such as gestational hypertension and pre-eclampsia, which usually present with choroidal thickening and hyperperfusion. Nevertheless, further prospective multi-center studies in uncomplicated, but also complicated, pregnancies with a larger number of subjects will be necessary to corroborate the findings of our proof-of-concept study. This might be useful for a better understanding of ocular and systemic diseases associated with pregnancy.

## Figures and Tables

**Table 1:** Demographic and clinical data.

	Pregnant Group	Control Group	*p*
Eyes	26 eyes (26 subjects)	26 eyes (26 subjects)	/
Age (years)	29 (22–35)	29 (21–35)	0.501
Gestational age (weeks)	35 (32–40)	/	/
MAP (mmHg)	93 (80–112)	89 (79–111)	0.115
AL (mm)	23.23 (22.18–25.69)	23.75 (22.25–25.64)	0.067
SE (D)	−0.25 (−2.630–1.630)	−0.25 (−2.750–1.875)	0.300
OCTA image quality (1–10)	10 (8–10)	10 (9–10)	0.365
BCVA (logMAR)	0.0 (0.0–0.1)	0.0 (0.0–0.1)	0.423
IOP (mmHg)	15.5 (10–21)	16 (12–20)	0.114

AL, axial length; BCVA, best-corrected visual acuity; D, diopters; IOP, intraocular pressure; logMAR, logarithm of the minimal angle of resolution; MAP, mean arterial pressure; OCTA, optical coherence tomography angiography; SE, spherical equivalent. Results are reported as median (range), and *p* < 0.05 was considered statistically significant.

**Table 2:** Choroidal metrics.

	Pregnant Group	Control Group	*p*
SFCT (µm)	332 (211–469)	371.5 (224–466)	**0.018**
CCP (%)	46 (41–50)	48 (41–52)	**0.039**
SLP (%)	62 (50–66)	61.5 (57–67)	0.839
HLP (%)	63 (48–69)	64 (58–68)	0.612

CCP, choriocapillaris perfusion; HLP, Haller’s layer perfusion; SLP, Sattler’s layer perfusion; SFCT, subfoveal choroidal thickness. Results are reported as median (range), and *p* < 0.05 was considered statistically significant.

**Table 3:** Correlation analysis of the control group.

		MAP	SFCT	CCP	SLP	HLP
MAP	cc	1	−0.209	0.291	0.044	0.430
	p	-	0.305	0.150	0.830	**0.028**
SFCT	cc	−0.209	1	−0.256	−0.177	−0.121
	p	0.305	-	0.207	0.386	0.555
CCP	cc	0.291	−0.256	1	−0.365	0.258
	p	0.150	0.207	-	0.067	0.203
SLP	cc	0.044	−0.177	−0.365	1	0.134
	p	0.830	0.386	0.067	-	0.516
HLP	cc	0.430	−0.121	0.258	0.134	1
	p	**0.028**	0.555	0.203	0.516	-

CC, correlation coefficient; CCP, choriocapillaris perfusion; HLP, Haller’s layer perfusion; MAP, mean arterial pressure; SLP, Sattler’s layer perfusion; SFCT, subfoveal choroidal thickness. Results are reported as median (range), and *p* < 0.05 was considered statistically significant.

**Table 4:** Correlation analysis of the pregnant group.

		MAP	SFCT	CCP	SLP	HLP
MAP	cc	1	−0.009	0.175	0.272	0.054
	p	-	0.965	0.392	0.178	0.792
SFCT	cc	−0.009	1	0.148	0.223	0.457
	p	0.965	-	0.471	0.273	**0.019**
CCP	cc	0.175	0.148	1	0.580	0.453
	p	0.392	0.471	-	**0.002**	**0.020**
SLP	cc	0.272	0.223	0.580	1	0.503
	p	0.178	0.273	**0.002**	-	**0.009**
HLP	cc	0.054	0.457	0.453	0.503	1
	p	0.792	**0.019**	**0.020**	**0.009**	-

CC, correlation coefficient; CCP, choriocapillaris perfusion; HLP, Haller’s layer perfusion; MAP, mean arterial pressure; SLP, Sattler’s layer perfusion; SFCT, subfoveal choroidal thickness. Results are reported as median (range), and *p* < 0.05 was considered statistically significant.

## Data Availability

Original datasets can be found at the Department of Ophthalmology, University of Lübeck.

## References

[1] Morton A. (2021). Physiological Changes and Cardiovascular Investigations in Pregnancy. Hear. Lung Circ..

[2] Osol G., Ko N.L., Mandalà M. (2019). Plasticity of the Maternal Vasculature During Pregnancy. Annu. Rev. Physiol..

[3] Schock H., Zeleniuch-Jacquotte A., Lundin E., Grankvist K., Lakso H., Idahl A., Lehtinen M., Surcel H.-M., Fortner R.T. (2016). Hormone concentrations throughout uncomplicated pregnancies: A longitudinal study. BMC Pregnancy Childbirth.

[4] Kur J., Newman E.A., Chan-Ling T. (2012). Cellular and physiological mechanisms underlying blood flow regulation in the retina and choroid in health and disease. Prog. Retin. Eye Res..

[5] Mackensen F., Paulus W.E., Max R., Ness T. (2014). Ocular Changes During Pregnancy. Dtsch. Ärzteblatt Int..

[6] Spaide R.F., Fujimoto J.G., Waheed N.K., Sadda S.R., Staurenghi G. (2018). Optical coherence tomography angiography. Prog. Retin. Eye Res..

[7] Spaide R.F., Koizumi H., Pozonni M.C. (2008). Enhanced Depth Imaging Spectral-Domain Optical Coherence Tomography. Am. J. Ophthalmol..

[8] Lauermann J.L., Sochurek J.A.M., Plöttner P., Alten F., Kasten M., Prasuhn J., Brüggemann N., Ranjbar M. (2021). Applicability of optical coherence tomography angiography (OCTA) imaging in Parkinson’s disease. Sci. Rep..

[9] Laviers H., Zambarakji H. (2014). Enhanced depth imaging-OCT of the choroid: A review of the current literature. Graefe’s Arch. Clin. Exp. Ophthalmol..

[10] Kashani A.H., Chen C.-L., Gahm J.K., Zheng F., Richter G.M., Rosenfeld P.J., Shi Y., Wang R.K. (2017). Optical coherence tomography angiography: A comprehensive review of current methods and clinical applications. Prog. Retin. Eye Res..

[11] Siegfried F., Rommel F., Rothe M., Brinkmann M.P., Sochurek J.A.M., Freitag J., Grisanti S., Ranjbar M. (2019). Evaluating diurnal changes in choroidal sublayer perfusion using optical coherence tomography angiography. Acta Ophthalmol..

[12] Barteselli G., Chhablani J., El-Emam S., Wang H., Chuang J., Kozak I., Cheng L., Bartsch D.-U., Freeman W.R. (2012). Choroidal Volume Variations with Age, Axial Length, and Sex in Healthy Subjects: A Three-Dimensional Analysis. Ophthalmology.

[13] Roskal-Wałek J., Laudańska-Olszewska I., Biskup M., Gierada M., Odrobina D. (2017). Choroidal Thickness in Women with Uncomplicated Pregnancy: Literature Review. BioMed Res. Int..

[14] Su L., Taweebanjongsin W., Gaw S.L., Rabina G., Sadda S.R., Tsui I. (2020). Evaluation of the Choroid in Women with Uncomplicated Pregnancy. Transl. Vis. Sci. Technol..

[15] Ranjbar M., Rothe M., Klapa S., Lange T., Prasuhn M., Grisanti S., Riemekasten G., Humrich J.Y. (2019). Evaluation of choroidal substructure perfusion in patients affected by systemic sclerosis: An optical coherence tomography angiography study. Scand. J. Rheumatol..

[16] Rommel F., Siegfried F., Sochurek J.A.M., Rothe M., Brinkmann M.P., Kurz M., Prasuhn M., Grisanti S., Ranjbar M. (2019). Mapping diurnal variations in choroidal sublayer perfusion in patients with idiopathic epiretinal membrane: An optical coherence tomography angiography study. Int. J. Retin. Vitr..

[17] Borrelli E., Parravano M., Sacconi R., Costanzo E., Querques L., Vella G., Bandello F., Querques G. (2020). Guidelines on Optical Coherence Tomography Angiography Imaging: 2020 Focused Update. Ophthalmol. Ther..

[18] Campbell I., Coudrillier B., Ethier C.R. (2014). Biomechanics of the Posterior Eye: A Critical Role in Health and Disease. J. Biomech. Eng..

[19] Ferrara M., Lugano G., Sandinha M.T., Kearns V.R., Geraghty B., Steel D.H.W. (2021). Biomechanical properties of retina and choroid: A comprehensive review of techniques and translational relevance. Eye.

[20] Azuma K., Okubo A., Suzuki T., Igarashi N., Nomura Y., Soga H., Murata H., Fujino R., Ogawa A., Matsui H. (2021). Assessment of the choroidal structure in pregnant women in the first trimester. Sci. Rep..

[21] Centofanti M., Migliardi R., Bonini S., Manni G., Bucci M., Pesavento C., Amin C., Harris A. (2002). Pulsatile Ocular Blood Flow during Pregnancy. Eur. J. Ophthalmol..

[22] Kara N., Sayin N., Pirhan D., Vural A.D., Araz-Ersan H.B., Tekirdag A.I., Yildirim G.Y., Gulac B., Yilmaz G. (2014). Evaluation of Subfoveal Choroidal Thickness in Pregnant Women Using Enhanced Depth Imaging Optical Coherence Tomography. Curr. Eye Res..

[23] Dadaci Z., Alptekin H., Acir N.O., Borazan M. (2015). Changes in choroidal thickness during pregnancy detected by enhanced depth imaging optical coherence tomography. Br. J. Ophthalmol..

[24] Nuzzi R., Scalabrin S., Becco A., Panzica G. (2018). Gonadal Hormones and Retinal Disorders: A Review. Front. Endocrinol..

[25] Viana L.C., Faria M., Pettersen H., Sampaio M., Geber S. (2011). Menstrual phase-related differences in the pulsatility index on the central retinal artery suggest an oestrogen vasodilatation effect that antagonizes with progesterone. Arch. Gynecol. Obstet..

[26] Rabiolo A., Gelormini F., Sacconi R., Cicinelli M.V., Triolo G., Bettin P., Nouri-Mahdavi K., Bandello F., Querques G. (2018). Comparison of methods to quantify macular and peripapillary vessel density in optical coherence tomography angiography. PLoS ONE.

[27] Chanwimol K., Balasubramanian S., Nassisi M., Gaw S.L., Janzen C., Sarraf D., Sadda S.R., Tsui I. (2019). Retinal Vascular Changes During Pregnancy Detected With Optical Coherence Tomography Angiography. Investig. Opthalmol. Vis. Sci..

[28] Khalil R.A. (2005). Sex Hormones as Potential Modulators of Vascular Function in Hypertension. Hypertension.

[29] Nesper P.L., Lee H.E., Fayed A.E., Schwartz G.W., Yu F., Fawzi A.A. (2019). Hemodynamic Response of the Three Macular Capillary Plexuses in Dark Adaptation and Flicker Stimulation Using Optical Coherence Tomography Angiography. Investig. Opthalmol. Vis. Sci..

[30] Nethery E., Brauer M., Janssen P. (2008). Time–activity patterns of pregnant women and changes during the course of pregnancy. J. Expo. Sci. Environ. Epidemiol..

